# What do medical students and their clinical preceptors understand by primary health care in South Africa? A qualitative study

**DOI:** 10.1186/s12909-023-04751-x

**Published:** 2023-10-20

**Authors:** Langalibalele Honey Mabuza, Mosa Moshabela

**Affiliations:** 1 School of Medicine, Clinical Integrated Programs, Sefako Makgatho Health Sciences University, 0012 Pretoria, South Africa; 2 Research and Innovation, University of KwaZulu-Natal, 4001 Durban, South Africa

**Keywords:** Primary health care, First contact healthcare, Comprehensive care, Continuity of care, Coordination of care, Undergraduate medical students, Clinical preceptors, Generalists, Specialists

## Abstract

**Background:**

The definition of Primary Health Care (PHC) issued by the World Health Organisation in 1978 indicated that essential health care should be made accessible to individuals and their communities close to where they live and work. In 1992 Starfield articulated the four pillars of PHC: the patient’s first contact with healthcare, comprehensive care, coordinated care and continuous care. Using this literature guidance, this study sought to explore what undergraduate medical students and their clinical preceptors understood by PHC in four South African medical schools.

**Methods:**

A qualitative study using the phenomenological design was conducted among undergraduate medical students and their clinical preceptors. The setting was four medical schools in South Africa (Sefako Makgatho Health Sciences University, Walter Sisulu University and the University of KwaZulu-Natal and the Witwatersrand University). A total of 27 in-depth interviews were conducted among the clinical preceptors and 16 focus group discussions among the students who were in their clinical years of training (MBChB 4–6). Interviews were digitally recorded and transcribed verbatim, followed by thematic data analysis using the MAXQDA 2020 (Analytics Pro) software.

**Results:**

Four themes were identified in which there were similarities between the students and their preceptors regarding their understanding of PHC: (1) PHC as the patient’s first contact with the healthcare system; (2) comprehensive care; (3) coordination of care and (4) continuity of care. A further two themes were identified in which these two groups were not of similar understanding: (5) PHC as a level or an approach to healthcare and (6) the role of specialist clinical preceptors in PHC.

**Conclusions:**

Medical students and their clinical preceptors displayed an understanding of PHC in line with four pillars articulated by Starfield and the WHO definition of PHC. However, there remains areas of divergence, on which the medical schools should follow the guidance provided by the WHO and Starfield for a holistic understanding of PHC.

## Background

In September 1978, in Alma Ata, the World Health Organisation (WHO) defined primary health care (PHC) as “the essential health care based on practical, scientifically sound and socially acceptable methods and technology made universally accessible to individuals and families in the community through their full participation and at a cost that the community and country can afford to maintain … in the spirit of self-reliance and self-determination” [1]. It was also described as the first level of healthcare contact for individuals and their families, bringing healthcare to where people live, constituting the first element of a continuing healthcare process [1].

The “first level of contact” refers to the principle of the management of an undifferentiated patient. This is a call for healthcare practice and student training in all health sectors to emphasise first-contact patient presentation [2]. A “continuing healthcare process” addresses the follow-up care in patient management. Health care clinical preceptors and their students should engage on how to conduct follow-up care of all patients following the initial clinical encounter, their referral to other levels of care and their receipt when they are down-referred to the institutions that initially referred them [3].

In 1992 Barbara Starfield articulated four cardinal pillars of PHC, namely (1) first contact care, (2) continuity of care, (3) comprehensive and (4) coordination of care, [4] which she and other authors have since elaborated on to incorporate further principles, namely person and family centered, equitable, team based and collaborative, integrated, accessible and of high value [5–7]. In 2018, the WHO broadened the scope of PHC even further as a whole-of-society approach to health, incorporating health promotion, disease prevention, curative, rehabilitative and palliative care throughout the life of individuals and communities [8].

In light of the increasingly complex nature of patient wellness and community health due to factors such as global migration [9] and inequity of resources [10], the expansion of PHC as defined by WHO bears relevance on “comprehensive care,” which deals with the holistic approach to patient care, addressing the biomedical, psychological and social dimensions of health and well-being (bio-psycho-social) [2] and “coordinated care” which focuses on the macro level system integration, putting the individual needs at the heart of the system in order to meet the needs of the population, because “what is best for individuals within a population is [also] best for the population” [2]. The complexity of patient care requires that PHC extends beyond health care into social care, requiring interprofessional and intersectoral collaboration to meet this challenge. According to the WHO, interprofessional collaboration occurs when “multiple health workers from different professional backgrounds work together with patients, families, carers and communities to deliver the highest quality of care across settings” [11]. This definition is pertinent in the context of medical student training in PHC as their training is facilitated by various professionals within the healthcare sector, e.g., nurse professionals. Intersectoral collaboration was explained by Kriesel as “a recognised relationship between part or parts of the health sector with parts of another sector which has been formed to take action on an issue to achieve health outcomes in a way that is more effective, efficient or sustainable than could be achieved by the health sector acting alone” [12], e.g., involvement of legal personnel in medical student training on medico-legal matters.

In line with the WHO definition of PHC at Alma Ata, South Africa adopted it as the organising principle of her national health systems and employed the district health system (DHS) as the vehicle for PHC delivery to communities [13].

Studies conducted in South Africa regarding the understanding of PHC have reported on the roles of clinicians in strengthening PHC [14]. Family physicians’ implementation of evidence-based practice in its capacity to guide PHC has been found to be limited. This may be attributed to their experiences and understanding of evidence-based practice and the implementation of evidence-based guidelines in PHC settings [15]. Regarding students’ understanding of PHC, studies have indicated that they understood PHC as a population perspective of health [16], continuity of care, a holistic approach to health care, accessible health, and health promotion [17]. A study on students’ perceptions of the applicability of the PHC approach in the South African context revealed that students were of the view that the PHC principles they were taught at the medical school environment may not be applicable in other settings in the country. They asserted that they could find themselves working in a system that would be different in terms of infrastructure and other resources, particularly in the under-resourced rural areas of the country [18]. A study which informed our study was conducted by Sibiya and Gwele in KwaZulu Natal in 2009, who analysed the meaning of integrated PHC among policymakers and coordinators of PHC at national, provincial and district levels, including PHC nurses at healthcare facility level [19]. One of the questions the participants were asked enquired on what they understood by “integrated PHC,” and the study findings were that PHC was conceptualised as comprehensive health care, employing a “supermarket” and a “one-stop shop” approach in healthcare delivery [19].

The importance of training medical students in PHC preparing them for the field of practice has been demonstrated [20]. In South Africa, there is evidence that undergraduate medical students do receive training in PHC [21, 22]. While the focus is on exposure to PHC [23], and students’ perspectives on its implementation [18], the understanding of PHC by both the students and their clinical preceptors has not been explored. However, its importance in establishing their position in this regard cannot be under-estimated as literature has shown that practice is guided by understanding [24]. This study sought to explore the understanding of medical students and their clinical preceptors in four South African medical schools, regarding their understanding of PHC in terms of the four pillars of Starfield [4], and the subsequent additional principles derived from the WHO definition of PHC (PHC as an approach to healthcare, and the role of specialists in the spectrum of PHC delivery) [8]. The findings of this study could inform undergraduate medical educators and policy makers on the current position of medical students and their preceptors in their understanding of PHC in South African medical schools.

## Methods

### Study context

This manuscript is part of the principal researcher’s PhD project, the overall aim of which was to explore the training of undergraduate medical students in general medical practice and PHC in four South African medical schools. Four manuscripts have been prepared, each reporting on one of the four study objectives, exploring (1) what the undergraduate medical students and their clinical preceptors understood by “general medical practice”; (2) what the students and their clinical preceptors understood by “primary health care”; (3) the experiences of the students and their clinical preceptors with respect to training in general medical practice; and lastly (4) the experiences of the students and their clinical preceptors in primary health care training. This manuscript reports specifically on the second objective.

### Researcher’s positionality

The principal researcher is a family physician by specialisation and a clinical preceptor for both undergraduate and postgraduate medical students. He is involved in the training of undergraduate medical students on PHC, together with other student preceptors. He is based at SMU, one of the institutions where the study was conducted. During data collection, at the beginning of each interview session he declared this positionality to the participants. During data analysis and interpretation, he was conscious of this background and maintained a reflexive approach to his worldview (entomological assumptions) [25].

### Study design

A qualitative study using the phenomenological design advocated by Terre Blanche et al. [26], was used to explore the understanding of PHC by undergraduate medical students and their clinical preceptors. The researchers regarded this design as appropriate since phenomenology is an approach that focuses on the lived experiences within a particular group [27]. Therefore, interviews were conducted with the undergraduate medical students and their clinical preceptors who had first-hand experience of working in a PHC environment.

### Study setting

This study was conducted in four of the nine medical schools in South Africa. An invitation had been extended to all the South African medical schools, but only Walter Sisulu University (WSU), Sefako Makgatho Health Sciences University (SMU) and the University of the Witwatersrand (WITS) accepted the invitation from the University of KwaZulu-Natal (UKZN) which received a grant for the Transformation in Medical Education (TiME) study [28]. Each medical school’s geographical location represented the country’s diverse geopolitical spectrum: WSU mainly rural, UKZN mainly rural with an urban component, SMU mainly urban with a rural component, and WITS mainly urban [29]. This varying geopolitical spectrum could have an influence on student exposure to the health facilities where their training in PHC took place [30].

### Data collection

Regarding the clinical preceptors, in-depth, one-on-one interviews were conducted by the research team (principal researcher and research assistant) at an interview venue determined by the participants. Participation was voluntary. The clinical preceptors comprised purposively selected heads of departments of disciplines or their delegates, e.g., course coordinators. The exploratory statement to open discussions for each student trainer was: “As a trainer of undergraduate medical students, what is your understanding of primary health care?” A total of 27 student clinical preceptors were interviewed in the four medical schools (Appendix A). Data were collected by means of an interview guide (Appendix B) derived from the study’s aim and objectives. Each interview lasted from 30 to 55 min. Regarding the medical students (Appendix C), focus group discussions (FGDs) were arranged through the students’ class leadership among students in their clinical years (MBChB 4–6). As recommended by Fern in collecting data among groups, each FGD comprised five to eight medical students [31]. Four FGDs were arranged per medical school: three homogeneous FGDs of students in the same year of study (4th, 5th and 6th year groups), plus one heterogeneous group comprising a mixture of students from the 4th, 5th and 6th years of study (about two students per year group, per FGD). The exploratory statement to open the discussion for each FGD among the students was: “As undergraduate medical students in this institution, what would you say is your understanding of primary health care?” A total of 16 FGDs (involving 102 students) were conducted in the four medical schools (Appendix C). The students’ FGDs were conducted at a venue chosen by the students and lasted for almost the same amount of time as those with their clinical preceptors. All interviews were conducted in English. An audio recorder was used to capture the interview discussions. The research team listened to each recorded interview on the day it was conducted and compiled reflective summaries to identify themes for both the clinical preceptors and the students.

Data collection was undertaken from 2016 to 2020 at the participating medical schools: WSU (2016), UKZN (2018 and 2019), SMU and WITS (2020). The initial interview process was through face-to-face contact with the participants. However, from 26 to 2020, the research team switched over to online interviews (via Zoom) in compliance with the COVID-19 regulations subsequently introduced by the South African government to curb the spread of the pandemic [32]. The switch-over only affected the data collection at SMU and WITS and only among the students, as all interviews with the clinical preceptors had been completed face-to-face before the pandemic. The principal researcher purchased one gigabyte (1G) of data for each participating student to ensure their expense-free participation.

### Trustworthiness

Trustworthiness of the study was ensured by consideration of credibility, dependability, confirmability and transferability of the study, as recommended [33]. Credibility, the extent to which the researcher’s findings are congruent with reality [34], was ensured by participant validation, whereby a dataset was created [Appendix D], containing the study data and analyses for participants’ feedback. Dependability, the reproducibility and stability of data [35], were ensured by a full description of the methods used in the data collection. However, variability was expected in this qualitative study as the focus was on the range of knowledge and experiences (some conflictual) rather than the average experience [36]. Confirmability, the objectivity of the researcher in data collection and reporting [37], was ensured by reflexivity, whereby the researcher was conscious of his influence on the participants as a trainer of medical students and allowed room for independent expression among all participants. Transferability, the degree to which the study conclusions can be applied to other similar settings [33], was ensured by providing sufficient data description to the reader [34]. During the interviews, field notes were taken and the students’ training manuals from each medical school were obtained for information triangulation [37].

### Data analysis

All recorded interviews were transcribed verbatim by a team of transcribers with expertise in linguistics. Each transcript formed the basis of data analysis. The principal researcher used both the inductive and deductive methods of data analysis [38]. As indicated in the interview guide (Appendix B), there was an unstructured exploratory question for both the one-on-one and FGD interviews which gathered broad information on the understanding of PHC, for which the inductive analysis method was used [34]. This was followed by open-ended prompts as a follow-up enquiry on matters which a participant had not initially address when responding to the broad exploratory question. For the responses to the semi-structured prompts, the deductive method was used [39]. The last prompt, which enquired on “any other comment regarding the participant’s understanding on PHC” was also analysed through the inductive method [38]. The MAXQDA 2020 (Analytics Pro) software program was used to arrange the data into data segments, categories, sub-themes and themes. The data segments obtained through the use of the program were 2179.

### Theoretical frameworks

This study made use of two theoretical frameworks: Vygotsky’s Social Constructivist theoretical framework developed by Lev Vygotsky in 1934 [40], and the Situated Learning theory, derived from the former by Lave and Wegner in 1991 [41, 42]. Both theories argue that learning occurs best when it takes place in the context in which it is applied. In the learning setting, there are apprentices (“novices”) who need facilitation by experts (the “more knowledgeable others”) to become experts themselves.

In the current study the theoretical frameworks served as a “map”, guiding the exploration of the medical students and their clinical preceptors’ understanding of PHC [43]. The exploration of the phenomenon did not focus on the participants’ awareness of the theoretical frameworks, but on their application as a tool to navigate the data, identifying the codes, categories and themes from the perspectives of both the “novices” and the “more knowledgeable others” regarding their understanding of PHC [44]. We followed the recommended Consolidated Criteria for Reporting Qualitative research (COREQ) for interviews and focus groups in reporting the study findings [45].

## Results

We identified six major themes for both the UG medical students and their clinical preceptors on their understanding of PHC, four of which were pre-determined by the interview guide prompts: first contact with the healthcare system, comprehensive care, coordination of care, and continuity of care; and two emerged unprompted: PHC as a level or an approach and specialist involvement in PHC. Some major themes had sub-themes (see Table 1).

**Table 1:** Themes and subthemes in the understanding of PHC by students and their trainers

Students	Clinical preceptors
Themes and sub-themes 1. First contact with the healthcare system 1.1. Accessible essential services for undifferentiated patients 1.2. Health promotion and disease prevention	Themes and sub-themes 1. First encounter with the healthcare system 1.1. Common medical conditions 1.2. Health promotion and disease prevention
2. Comprehensive care 2.1. Social determinants of health 2.2. Patient advocacy	2. Comprehensive care 2.1. Biopsychosocial approach 2.2. Social determinants of health 2.3. Patient advocacy
3. **Coordination of care**	3. **Coordination of care**
4. **Continuity of care**	4. **Continuity of care**
5. **PHC – a level or an approach to healthcare?** 5.1. A level of care 5.2. It is both	5 **PHC – a level or an approach to care?** 5.1. A level of care 5.2. An approach to care
6. **Specialists’ involvement in PHC** 6.1. They should be involved 6.2. Uncertainty	6. **Specialist involvement in PHC** 6.1. They should be involved 6.2. They should not be involved

## Students’ understanding of PHC

### First point of contact with the health care system

The most frequently mentioned understanding of PHC by students was that it was the first point of patient contact with the healthcare system which was mainly rendered in community facilities. The students understood the community facilities as PHC training platforms. To this end, the students felt that training at tertiary institutions took them away from these platforms.

*So, primary health care is basically the first contact that patients go to, which means the clinic, the primary health care, community health care centres and then I’d also say the district hospitals including the GPs, they’re all primary health care …* (KZS5.2, MBChB 5, male students, 22 years).

### Accessible essential services for undifferentiated patients

The students elaborated on PHC as the first point of patient contact with the health care system by describing it as a forum where accessible essential services are rendered to mostly undifferentiated patients. These patients may need escalation to higher levels of care if they have complications.

*What I understand about primary healthcare, one of the essential things about primary healthcare, is the provision of essential services, and essential treatment.* (WTS6.1, MBBCh 6, male student, 23 years). *Yes, it is for everyone, and it must be accessible to everyone in the community. I know that that’s the component of Alma-Ata.* (SMSM6.1, MBChB 6, male student, 24 years).

*So, like my colleague said, you’re going to be referred to someone who has more experience and who’s more specialized in that field if they can’t take care of you. So, it’s where [at primary health care] patients who don’t have complications are actually helped.* (WTS5.1, MBBCh 5, male student, 22 years).

### Health promotion and disease prevention

Students understood health promotion and disease prevention to be rendered mainly at PHC platforms, in contrast to specialist platforms.

*Primary health care is about health promotion. Going out to the, to the communities and teaching people about ways to prevent… prevent them from getting sick … teaching them how to lead a healthy lifestyle, from exercising, to healthy diet, just adopting healthy habits.* (KZSM5.2, MBChB 5, male student, 22 years).

*I think that it is a health care system that focusses on prevention of disease, health promotion that other specialists don’t deal with, [as] they’re more curative than preventative.* (KZS6.1, MBChB 6, male student, 22 years).

## Comprehensive care

Some students mentioned that PHC was comprehensive care in that it took into consideration not only the patient’s disease, but their contexts as well. This was explained in terms of the social determinants of health (availability of basic resources) and patient advocacy (empowering the patient with knowledge regarding their conditions, thus enabling them to assume responsibility in the management).

### Social determinants of health

There was demonstration of understanding of social determinants of health.

*With social determinants of health, with what we were being taught, it includes things like where the patient is staying, where the patient is employed, and also let’s say the rural area, where they get water….* (SMS5.3, MBChB 5, male student, 41 years).

### Patient advocacy

This was understood to be achievable through patient empowerment with knowledge.

*Like teaching a patient to use an asthma pump inhaler. You actually have to demonstrate it. Guide the patient through it step-by-step. So, that gives the patient empowerment and knowledge and the patient will be able to deal with their condition better and will be able to take charge [of their conditions].* (SMS4.1, MBChB 4, female student, 22 years).

### Coordination of care

In relation to their understanding of PHC, students understood coordination of care as when a PHC practitioner with a general medical approach takes leadership in ensuring the collaboration of the various disciplines to optimize patient care.

*So, basically it means that this general practitioner he’s able to work together and optimize all other members of the medical team in order to ensure that the best approach is actually achieved for that patient*. (KZSM5.1, MBChB 5, female student, 23 years).

### Continuity of care

Continuity of patient care, which was understood as a component of PHC, was understood as ensuring a continuing relationship with a patient, which entailed physical patient follow-up at health facilities and patients’ homes and also by phone calls.

*So, continuity of care is seeing your patient on a… like following up on your patients, … when you do that you form a relationship with them as a doctor, … you have patients who do go regularly for their treatments for diabetes or hypertension. Some doctors … also go to their patients houses*, (WTS5.5, MBChB 5, male student, 23 years).

### PHC – an approach or a level to health care?

There were different views on whether PHC should be understood as a level, (connoting that it was solely applicable at lower levels of the health care delivery system at PHC settings like clinics and district hospitals); or an approach to care (implying that it could be practiced at all levels of the healthcare delivery system – primary, secondary, tertiary and quaternary levels), or both.

### A level of care

*Like primary health care from what I understand it to be, is a level. So, you have primary health care and then that can be further escalated in[to] secondary institutions and then tertiary institutions.* (WTSM4.1, MBBCh 4, female student, 20 years).

### It is both

*I feel like maybe it [primary health care] can be both [an approach and a level]. [It] doesn’t mean that the approach used at the primary healthcare level can’t be applied later on [referring to higher levels].* (WTSM6.1, MBChB 6, female student, 23 years).

### Specialists’ involvement in PHC

There were differences of opinion among the students on whether specialists should be involved in PHC training, with some holding the view that specialists should be involved while others expressing their doubt.

### They should be involved

*So, I remember there was a concept of upstream when we are seeing patients from a certain community with a certain condition, we [were told] we should look at the upstream factors, what is causing these conditions and then focus on prevention and awareness etc. So, I think any other discipline can adopt that and then just try to stop the problem before it happens - at prevention.* (KZSM4.2, MBChB 4, male student, 21 years).

### Uncertainty

… *I’m not sure, but I think people who are specialists, like surgeons or whatever, they deal with things that have already passed from a general practitioner. So, basically a surgeon can’t be the first person you go to. I’m not sure if they deal with primary health care, in what context?* (WTS4.1, MBBCh 4, male student, 23 years).

## Medical preceptors’ understanding of PHC

### First encounter with the health care system

Like their students, most clinical preceptors indicated that they understood PHC as the entry point to health care for patients presenting with any medical condition as well as the beginning point to health care. It was also understood in terms of the facilities where it is practised – at the basic community facilities as well as their actual involvement in those communities as clinicians.

*I think for me, the primary health care is the first point of entry in the health system. In other words, this is where every patient should start. So, it’s really the starting area, the kindergarten of health care,…* (BKZT5, clinical preceptor, General Surgery).

*Primary health care is the first point of contact which the patient has when they are having any illness, … and for most of us the primary health care would also be inclusive of facilities which are called primary health care.* (DWTT6, clinical preceptor, Paediatrics).

*I think for me, the primary health care is the first point of entry in the health system. In other words, this is where every patient should start.* (CSMT5, clinical preceptor, General Surgery).

### Common medical conditions

The understanding was also that, as the first point of patient contact, common medical problems in communities were attended to at PHC. The common medical conditions were explained as those that were uncomplicated and essential, which every student was required to be familiar with.

*So, I see primary healthcare as those kinds of knowledge and skills that’ll address a patient’s health needs at a point of care, which is in a primary district level. So basically, what I’m saying is that, when a patient wakes up with a problem, they’ll present to a health centre. That health centre should be able to provide at a primary healthcare setting, probably 80% of the patients’ needs, and refer everything else that they can’t do.* (DWTT4, clinical preceptor, Obstetrics and Gynaecology).

*They [students] are taken through wide array of conditions that we think that these are conditions that students need to know when they get out there [in the community].* (AWST4, clinical preceptor, General Surgery).

*To me that will be primary health care, you know, … making sure that my students are aware of the diagnosis of these particular disease entities. They must know everything about HIV, they must know everything about TB, they must know everything about hypertension, diabetes, asthma, COPD, thyroid. Those are things that we see on a daily basis.* (DWTT3, clinical preceptor, Internal Medicine).

### Health promotion and disease prevention

At the first point of patient contact, PHC was seen as the platform where health promotion and disease prevention (also described as screening) was practiced.

*Yah, I think really, the primary health care aspect should be about prevention of problems, picking up and screening for the ones that we know that, if left on their own, there are potential problems in our society.* (BKTZ4, clinical preceptor, Obstetrics and Gynaecology).

*That’s how I think primary health care should be. Primary [health care] honestly should be primary prevention.* (CSMT3, clinical preceptor, Internal Medicine)

## Comprehensive care

PHC was also understood as provision of comprehensive patient care.

### The biopsychosocial approach

It entailed attending to the biopsychosocial aspects of each patient encounter.

*That enlightens our students to tell them that this psychosocial history that you’re taking is very important … it tells you how you’re going to need to tailor your therapeutic interventions with respect to this patient, because then if a patient comes from a rural area in KwaZulu-Natal, you can’t put this patient on warfarin, because he can’t come for assessment of his regular INR [International Normalised Ratio].* (DWTT3, clinical preceptor, Internal Medicine).

*the students are [taught] to know how to integrate other medical related specialties into patient care, so that we can have a comprehensive approach to patient management.* (AWST4, clinical preceptor, General Surgery)

### Social determinants of health

Like the students, one clinical preceptors also brought into the picture the importance of social determinants of health. The social determinants of health were described as addressing patients’ social inequalities and alerting them on lifestyle modifications.

*But what we do know and what we do stress is that, when we’re doing our assessment or when we ask our students for an assessment, one of the main things is lifestyle modification. And what that entails – whether it’s just, you know, looking at… addressing the particular social inequalities that the patient might be exposed to, or whether it’s got to do with dietary habits, or whether it’s got to do with lack of exercise, are all brought in into that particular assessment* (DWTT3, clinical preceptor, Internal Medicine).

### Patient advocacy

Advocacy for health was understood as the interventions by a clinician to empower patients with knowledge and improve their conditions.

*… we kind of ask them to frame the intervention in terms of advocacy. So, we’re saying, if you are doing a health promotion project what are you advocating for? Who are you advocating for? What change do we need to see …? So, it’s like a lot more oriented around change than just the kind of, ‘we did a talk about diabetes in the waiting room.’* (BKZT1, clinical preceptor, Family Medicine).

### Coordination of care

One student clinical preceptors understood coordination of care as the practice of team-based health care. This was expressed by an obstetrician who indicated that coordination of care was for done for good patient care outcomes. general

*…so I started teaching nurses, eh… I started teaching clinical associates, but I’ll want to teach them in one room, so that they know that they are part of a team …. And I think if we do that, eh…the patient outcome will be much better because we will be teaching these guys that this is how care is provided; is not provided by an obstetrician without a nurse, you know, … we let them work together and learn how to communicate when they coordinate patient care?*

(DWTT4, clinical preceptor, Obstetrics and Gynaecology).

A family physician and a dermatologist also added their understanding of coordination of patient care in the medical team:

*So, all of us recognize that we [doctors] are not the only ones that are able to coordinate the care, … if somebody has got a car accident in the first instance, it’s the paramedic who is the coordinator of the care - they make the arrangements for the helicopter to come …. In the trauma unit it’s the trauma surgeon who is the coordinator of care till that person stabilizes or whatever. In [the] ICU it may be the trauma surgeon or the anesthetist, or whoever,… you know, and if that person has got an amputation, as they go home, it’s then the therapist who becomes the coordinator for care.* (BKZT1, clinical preceptor, Family Medicine).

*So that’s bringing in the teamwork, it comes up in their training but there are certain conditions even when you’re just a dermatologist you need to involve the other specialties*. (AWST6, clinical preceptor, Dermatology).

### Continuity of care

In relation to PHC, in one institution a student clinical preceptors understood continuity of care in terms of patient follow-up, from the point of admission to discharge. Students were allocated patients to take care of in line with this understanding.

*So, if a patient has moved from [the] emergency [unit] to your ward, it means you have to manage that patient from coming until they are discharged. So, then continuity of care is happening, and when you discharge you have to have a plan of discharge. What is going to happen with this patient at discharge? Am I seeing this patient back? Am I referring this patient down to the clinic?* (DWTT6, clinical preceptor, Paediatrics).

## PHC – a level or an approach to care?

The clinical preceptors also had varying views on whether PHC should be understood as an approach to or a level of care. A level was also referred to as a “place” (implying a setting like a clinic or a hospital), while an approach implied patient care, regardless of the level at which it was rendered. The former focuses on the categorisation of the facility while the latter on the centrality of the patient in the health service equation.

### A level

*We should be defining to say; okay, this we can’t do at this [PHC] level, … either because of limitation of resources, or because of the technical skills that they need.* (DWTT4, clinical preceptor, Obstetrics and Gynaecology).

*I think that it [PHC] is that level, that level of health care with the first point of contact, in most cases …* (CSMT1, clinical preceptor, Family Medicine).

### An approach

One trainer pointed out that students were getting confused when PHC was categorised in terms of levels based on health facility structures, because in private practice [in South Africa], these structural hierarchies do not exist – all patients are managed under one facility.

*So, for me, it’s two things – it’s the kind of service and it’s a place. And this debate comes when we go to private practice, which is where these guys are going [to work, after qualifying], and private tells you, we don’t have a district hospital in private, we have a hospital … we [the trainers] are disempowering generals because they think once you are in private, all children must be seen by a paediatrician and I’m challenging that notion.* (CSMT6, clinical preceptor, Paediatrics).

## Specialist involvement in PHC

The students’ clinical preceptors had divergent understanding on the involvement of specialists in PHC.

### They should be involved

Some felt specialists had a role to play in PHC – they could immunize children with missed doses, and they were involved in research that established primary causes of conditions.

*…you are a general before you become a specialist, and as a specialist, … there’s nothing that says I cannot practice primary health care. By level they would say immunizations happen at the clinic, isn’t it? And that will make it a primary health care function. But when they come to me [a specialist], I would advocate and say: ‘In my hospital if a child has not been immunized fully, can I advocate to actually give those missed immunizations.’* (DWTT6, clinical preceptor, Paediatrics).

Another motivating view for specialist participation in student training on PHC came from one institution where it was acknowledged that they conduct outreach training activities for students at distributed training sites.

*Our district hospitals are level one hospitals, you don’t have longitudinal specialist [trainers] there, [students] are taught there by the generalists, but the university on a regular basis all the departments make the major departments send consultants and registers to cover agreed upon topics from their specialties.* (AWST1, clinical preceptor, Family Medicine)

A study was quoted where specialist surgeons brought about improved healthcare through PHC principles of dealing with the root cause of conditions.

S*o, do we [specialists] play a role in prevention of disease? Well, I think ‘Yes!’. I think if we take some of the things that I’ve already spoken about, burns and prevention of burns, road traffic accidents - I mean there’s a wonderful study that was done in Ghana, where they [surgeons] looked at one particular road, they looked at the motor vehicle accidents and they looked at the people and the mortality rate from that, and then they wrote a report saying seatbelts were needed and … mandatory seatbelts were implemented and then they looked at the morality rate, and the mortality rate went down* (DWTT5, clinical preceptor, General Surgery).

### They should not be involved

There was also a strong voice of dissension regarding the participation of specialists in PHC, given what they regarded as time constrains.

*We don’t talk about primary health care at all in Internal Medicine, because the primary health care is something that is happening outside of the medical ward round, … I mean the patients that are coming to us are critically ill - I would say 60–70% of the patients in my ward [at] any given point in time, if they were in a private sector they would be in high care or ICU. So, we’re seeing patients critically ill with advanced disease, with organ failure - the conversation is never around primary health care* (BKZT3, clinical preceptor, Internal Medicine).

*… we don’t focus on that particular aspect of the management [PHC] … the time frame that we have is too limited.* (DWTT3, clinical preceptor, Internal Medicine).

## Discussion

The study has demonstrated that students and their clinical preceptors shared a common understanding of PHC as the patient’s first point of contact with the healthcare system, comprehensive care, coordination of care and continuity of care. Both groups had various opinions on whether PHC was to be regarded as a level, an approach, or a combination of both towards health care. Some students expressed a view that specialists should be involved in PHC training while others were uncertain. There were also contrasting views among their clinical preceptors in this regard.

Both students and their clinical preceptors understood PHC as the first point of patient entry into the healthcare system, in keeping with the view held by the WHO [1] and Starfield’s pillars on PHC [4]. As the point of first patient contact, students indicated that PHC necessitated training in common and uncomplicated clinical conditions. Their clinical preceptors referred to these conditions as “any medical condition” to convey the idea of an “undifferentiated patient,” which characterizes PHC [46, 47]. Students also indicated that PHC was optimally practiced in community facilities, and consequently viewed their training mainly based in tertiary institutions as taking them away from the PHC training platforms. Indeed, the approach of tertiary institutions towards patient care has been reported as curative and facility-based, compared to community-based health care, which emphasizes disease prevention and health promotion [48]. The authors of this study are of the view that the students’ clinical preceptors have succeed to communicate the understanding of PHC as the point-of-first contact to their students in the four medical schools.

Some students mentioned that PHC was comprehensive care in that it took into consideration not only the patient’s disease, but their contexts as well. One student trainer used the term “the biopsychosocial aspect” to describe the samephenomenon. The University of Putra in Malaysia has demonstrated that medical education should incorporate both medical science and social science disciplines to equip students to understand the multifaceted nature of disease conditions [49]. At that university, students were trained to arrive at a patient diagnosis that was not only based on physical examination for clinical signs and symptoms, as that approach fell short of addressing the patient’s needs comprehensively. In this study, students also mentioned social determinants of health (SDH) as forming part of comprehensive care. Student clinical preceptors added the need for a clinician to attend to the patient’s lifestyle modifications which they viewed as linked to the SDH. There is literature evidence of this link particularly among the elderly [50]. Rasanathan et al., have already demonstrated that SDH and PHC are priorities in addressing health equity in communities [51]. Therefore, successful reduction of health inequities is achieved through intentional redress of SDH in communities. In mentioning SDH, students also indicated the need for patients to be empowered through patient advocacy. Patient advocacy was explained as empowerment of patients through education on their conditions, enabling them to be partners in the management. A recently conducted scoping review has shown that patient education is an effective tool in empowering patients with chronic diseases for self-management [52]. Based on the findings of this study, the understanding of PHC by both the students and their clinical preceptors augurs well for a good foundation in PHC training in the four South African medical schools.

Students understood coordination of care as when a PHC practitioner with a general medical approach takes leadership in ensuring the collaboration of various disciplines to optimize patient care. In keeping with the student’s view, the pivotal role of a general medical practitioner in the integration of multidisciplinary patient care has been described [53]. The student clinical preceptors understood coordination of care as the practice of team-based healthcare which was not solely reserved for the doctor – it could be any appropriate healthcare practitioner in a given clinical situation. The clinical preceptors believed that a coordinator should be decided on through collegial consensus. The position of the clinical preceptors is backed by a recent study conducted on 60 healthcare teams which demonstrated that in a multi-disciplinary healthcare team, the discipline with “the most pertinent expertise relative to the topic under discussion” should take leadership [54]. It is the view of the authors that the clinical preceptors in the four South African medical schools have the responsibility to alert their students on the importance of collaborative decision-making on team leadership to break down the historically entrenched hierarchies in medical care, whereby medical doctors’ decision-making processes were perceived unchallengeable [55]. Furthermore, the notion that was expressed by some students and preceptors, that medical specialists should not be involved in the training of medical students on PHC, negates this Starfield’s pillar on comprehensive patient care [4], which incorporates medical specialists’ involvement.

Students and their clinical preceptors understood continuity of care as the establishment of a continuing relationship with a patient, taking the responsibility of making follow-ups on the patient’s well-being beyond the first encounter. This informed the training methods students received: making follow-up enquiries when patients were up-referred to specialist care or when they were discharged home. There is acknowledgement that it is difficult to provide longitudinal care experiences among students, given the dynamic nature of the training platforms whereby patients are lost to follow-up for various reasons [56–58]. In a study that explored the views of patients who were allocated students to provide them with continuity of care, it was shown that patients valued the relationship with students similar to that described between patients and their qualified physicians [59]. The patients appreciated the role played by students in linking them up with their physicians, while the students also benefitted from the training in continuity of care [59]^59^. In our study, the dovetailing understanding of the continuity of patient care between the students and their clinical preceptors displayed the potential to set the scene for effective student training in that regard.

Regarding the understanding of PHC as a level of care or an approach to healthcare, divergent views were found among both the students and their trainers. The aspirations of PHC have been addressed in the WHO definition of PHC as “the whole-of-society approach in health, aimed at providing equitable health and well-being to individuals, families and communities, as early as possible in the continuum of health, namely health promotion, disease prevention, curative, rehabilitative and palliative care, as close as feasible to people’s day-to-day environments” [8]. In their understanding of PHC, the clinical preceptors and their students need to come to terms with this global definition of PHC by the WHO as an approach, not a level of health care, and reflect that understanding in student training on PHC. Furthermore, it was noted that neither the students nor their clinical preceptors alluded to the palliative care aspect of the WHO definition in their understanding of PHC. In 2004, the WHO made a recommendation to all governments to include palliative care in the curricula of health workers at all levels [60]. However, in Africa there is still shortage of doctors and nurses with adequate skills and training in palliative care [61], impacting on student training. Burger et al., in their recently published position paper on undergraduate palliative medicine education for doctors in South Africa, have remarked on the lack of consensus and standardisation of the content, structure and delivery of palliative training programmes in South Africa [62].

There were differences of opinion among the students on whether specialists should be involved in PHC training, with some holding the view that specialists should be involved while others expressing their doubt. The authors of this paper did not find studies which reported on medical students’ views on the appropriateness of the involvement of specialist clinical preceptors in PHC training in South Africa. However, there is a global move towards training medical students in PHC in distributed healthcare units [63], where the majority of the student clinical preceptors are generalist practitioners. Some specialist clinical preceptors felt they had a role to play in PHC, citing that they were capable of executing some PHC functions, like immunization of children. The preventive function of specialists has also been reported in Europe [64]. In one institution mention was made of the specialist departments sending outreach teams for student training at the distributed training sites. In South Africa specialists to whom students are mostly exposed in the distributed learning sites are family physicians [65–67], and rarely other specialists [68, 69]. However, one specialist clinical preceptor strongly believed that PHC was not in his territory, arguing that he had limited time to practice comprehensive patient care required for PHC. There is evidence suggesting that the solution to these divergent views between generalists and specialists could be the establishment of interdisciplinary collaborations [70]. The benefits of specialists’ inclusion in undergraduate medical student training have been demonstrated in many parts of the world [71–73]. The authors are of the view that the argument that specialists should not be involved in the PHC training of undergraduate medical students contradicts the WHO definition of PHC which includes “curative care” in the “the continuum of health” in which (curative care) specialists are mainly involved [8]. Further studies are needed in South Africa to explore opinions of medical students and their clinical preceptors regarding the role of specialists in PHC training.

### Study limitations

The limitation of this study is that it was conducted in only four of the nine medical schools in South Africa, even though all had been invited. Therefore, the study findings cannot be generalized with certainty to the other South African medical schools but could be transferrable under similar contexts. Furthermore, social desirability bias on the part of students was unavoidable [74], given that they were interviewed by the principal researcher who is himself a clinical preceptor. Therefore, in their participation, the students may have behaved in a manner they thought would be acceptable to him.

## Conclusion

Although the students and their clinical preceptors displayed an understanding of PHC as defined by Starfield’s four pillars and the WHO definition of PHC, there remains room for further understanding of PHC as a level of or an approach to healthcare, as well as the role of the specialist preceptors in the training of the students on PHC in South African medical schools. The guidance provided by the WHO definition on the spectrum of PHC and Starfield’s comprehensive pillar of PHC should be adhered to by these medical schools.

## Data Availability

The datasets (Appendices A-D), relating to list of participants (students and their trainers) and interview guide are available at: https://figshare.com/s/4f59b5082d6ee6efafb0.

## Abbreviations

FGD: Focus group discussion
PHC: primary health care
SDH: social determinants of health
MBChB: Bachelor of Medicine and Bachelor of Surgery
